# Problematic Internet Usage and Immune Function

**DOI:** 10.1371/journal.pone.0134538

**Published:** 2015-08-05

**Authors:** Phil Reed, Rebecca Vile, Lisa A. Osborne, Michela Romano, Roberto Truzoli

**Affiliations:** 1 Università degli Studi di Milano, Milan, Italy; 2 Abertawe Bro Morgannwg University Health Board, Swansea, United Kingdom; 3 Swansea University, Swansea, United Kingdom; University of Granada, SPAIN

## Abstract

Problematic internet use has been associated with a variety of psychological comorbidities, but it relationship with physical illness has not received the same degree of investigation. The current study surveyed 505 participants online, and asked about their levels of problematic internet usage (Internet Addiction Test), depression and anxiety (Hospital Anxiety and Depression Scales), social isolation (UCLA Loneliness Questionnaire), sleep problems (Pittsburgh Sleep Quality Index), and their current health – General Health Questionnaire (GHQ-28), and the Immune Function Questionnaire. The results demonstrated that around 30% of the sample displayed mild or worse levels of internet addiction, as measured by the IAT. Although there were differences in the purposes for which males and females used the internet, there were no differences in terms of levels of problematic usage between genders. The internet problems were strongly related to all of the other psychological variables such as depression, anxiety, social-isolation, and sleep problems. Internet addiction was also associated with reduced self-reported immune function, but not with the measure of general health (GHQ-28). This relationship between problematic internet use and reduced immune function was found to be independent of the impact of the co-morbidities. It is suggested that the negative relationship between level of problematic internet use and immune function may be mediated by levels of stress produced by such internet use, and subsequent sympathetic nervous activity, which related to immune-supressants, such as cortisol.

## Introduction

Excessive or maladaptive use of the internet (or problematic internet usage) has been suggested by some as problem for certain groups of individuals [1,2], and the need for further study concerning whether an Internet Addiction Disorder (IAD) is a useful concept has been suggested [1,3]. Individuals reporting problems concerning their internet usage note a number of associated symptoms, such as: major disruption to their work and social relationships [4,5,6], and negative affect when separated from the internet [7]. Estimates of the prevalence of problematic internet usage in the general population vary between 2% and 8%, and range up to 20% in younger samples [3, 8–10], although these figures are difficult to interpret precisely due to the differing definitions of ‘problematic internet usage’ or ‘internet addiction’ that are employed.

Those people who report problematic internet use also report a wide range of associated psychological and social problems [10–12]. Psychological co-morbidities noted in those individuals who report problematic internet usage have been found to include: anxiety [7,13,14], attention-deficit hyperactivity disorder [15], autism spectrum disorders [7,16], depression [13–15, 17], impulse dysregulation and hostility [18–20], and schizophrenia [7,21]. Social anxiety disorder [18] and loneliness [22], are also very commonly associated with IAD. In addition, high levels of life stress [23] and social isolation [22, 24–26], and a lower quality of life [24,27], are mentioned by those who report problematic internet use

Problematically high levels and types of internet usage also have been associated with neurological changes [28,29]. An increasing amount of research suggests that problematic internet use, in common with other behavioural addictions, is associated with abnormalities in the dopaminergic system [30,31], and with increased sympathetic nervous activity [32,33], which also have been shown to be related to one another [34].

In contrast to the growing literature regarding the psychological and neurological correlates of IAD, there have been few studies of the impact of problematic internet use on physical health. A relationship between disturbed sleep and heavy internet use has been established [35,36], as has a relationship between problematic internet use and a poor diet [37] resulting in weight problems, such as obesity [38]. Some research has found associations between problematic internet usage and self-reported health-related quality of life, a concept related to illness, although it should be noted that there are very few such demonstrations and there are discrepancies in this literature [39,40]. For example, health related quality of life, measured by the SF-36, has been found to correlate with problematic internet use, although quality of life does not correlate with time spent using the internet [40]. In contrast, when health-related quality of life has been measured by the General Health Questionnaire (GHQ), little relationship has been noted with IAD [9,39]. The reasons for the different patterns of findings using these two measures of health-related quality of life are unclear–although they may reflect both differences in the operationalization of the notion of problematic internet use across studies, and the focus of the SF-36 on both physical and psychological health-related quality of life compared to the primarily psychological-focus of the GHQ. Thus, the literature with respect to health-related quality of life is currently difficult to interpret.

The above discussion implies that further research in this potentially important area is warranted, given the increasing use of the internet [3], and the lack of clear evidence relating to its impact on health functioning *per se* as opposed to health-related quality of life, as well as the attendant problems that increased levels of associated physical illness may cause for health systems. Of course, given the co-morbidities exhibited by those who report problematic internet use, any relationship between problematic internet use and physical illness may be the product of any one of a number of issues. Neglect of the self by those reporting problematic internet use in terms of a poor diet and poor sleep patterns, may be involved in increased levels of physical illness [37,40]. Certainly, poor sleep has been shown to predict some aspects of immune function [41–43]. Additionally, the co-morbid psychological issues may also play a role. It has been noted that mental health problems are correlated with the number of colds reported during a year [44]. Specifically, both depression [45–47], and anxiety and stress issues [48], especially social anxiety and loneliness [49–51], predict immune dysfunction. Finally, activation of the sympathetic system, which is noted in those with problematic internet use, is correlated with increases in adrenaline and cortisol levels, and leads to decreased immune function, especially in those with high levels of reported stress [52]. Any investigation into the relationship of problematic internet use and physical illness will require some assessment of the relative contributions of these related aspects of functioning.

Obviously, physical health is a very wide-ranging concept, but the above review suggests that problematic internet usage might impact specifically on immune function, which has received no direct study [53]. If this is the case, then illnesses such as the common cold [54], influenza [55], cold sores [56], pneumonia [57], sepsis [58], and skin infections [59], may be key to focus upon when assessing the impact of problematic internet use on physical symptoms. As noted above, previous explorations of the relationship between problematic internet use and physical illness have tended to focus on reports of health-related quality of life obtained using instruments such as the SF-36 and the GHQ. Although these measures are reliable, they do not necessarily focus on any specific set of illnesses, and are not related to the illnesses that individuals with suppressed immune systems might be prone to exhibiting. In determining the degree to which immune function may be compromised, previous work has examined self-reports of the symptoms typically correlated with poor immune function [31,44]. Self-report is regarded as a strong method in this context, as such symptoms are easy to self-discriminate, are often not reported to health professionals and so do not show up on medical records, and are often experienced without any objectively verifiable viral cause [54].

Given these above considerations, the current study explored the relationship between problematic internet usage and two primary indictors of health (immune function and self-reported health status), as well as a range of health-related variables (depression, anxiety, loneliness, and sleep problems). Of particular interest was the relationship between problematic internet use and immune-related physical health, which has not previously been assessed specifically. In this regard, an initial aim of the study was to investigate whether higher levels of problematic internet use would be associated with greater report of immune-related illnesses (over and above the potential impact of the internet problems on the other health-related variables measured). In addition, there were a number of secondary aims that have not previously been examined of the study, including exploring the nature of the relationship between problematic internet use and self-reported health status. This was examined in order to determine whether this variable displayed the same relationship to problematic internet use as reports of immune-related symptoms. A range of other potentially associated problems for those displaying problematic internet use, that have also been found to predict poor immune function, such as anxiety, depression, loneliness, and sleep problems, were measured in an attempt to determine the relationship between problematic internet use and physical health symptoms independent of these co-morbid problems. This should allow a first step in establishing the nature of any relationship between problematic internet use and reduced immune function, should an association be found to exist.

## Method

### Ethical Statement

Ethical approval for this research was obtained from the Department of Psychology Ethics Committee, Swansea University. The participants provided informed consent to participate in this study by signing a consent form following reading the information sheet provided for them, and the Ethics Committee approved this consent procedure.

### Participants

Five hundred and five participants (265 females and 240 males) were recruited through links posted on internet sites (social networking sites, blogging and microblogging sites, and gaming sites). An online recruitment strategy was adopted in line with previous explorations of the impact of problematic internet use [60,61].

All participants were volunteers, and none received any form of compensation for their participation. The participants had a mean age of 29.73 (+ 13.65, range 18–101) years: < 20 years = 7.5%; 21–29 years = 61.8%; 30–39 years = 15.5%; 40–49 years = 4.6%; 50–59 years = 4.2%; 60+ years = 5.9%. The participants’ self-reported ethnicity was: 202 (40%) White; 50 (10%) Mixed/Multiple Ethnic Groups; 141 (28%) Asian; 106 (21%) Black/African/Caribbean; and 6 (1%) Other Ethnic Group. The marital status of the sample was: 305 (60%) single, 65 (13%) married or in a civil partnership; 105 (21%) in other forms of relationship; and 30 (6%) divorced or widowed.

### Participant’s Typical Use of the Internet

Participants were asked to estimate their average internet use by asking them to estimate the number of hours per week that they had spent on the internet over the last few months; this measure is commonly taken in studies of problematic internet use [40,61]. Although it has been suggested that it is ‘non-professional’ usage that correlates with several problems associated with heavy internet use [40], it was thought that the professional/non-professional distinction may not apply to all respondents, and that these usages might also be difficult to discriminate for some respondents. Moreover, total internet use, in itself, has also been found to be associated with internet-related problems [40].

The mean number of hours per week internet use reported was 39.57 (+ 28.06, range = 1 to 135): 28.3% reported spending between 1 and 20 hours per week online; 29.5% reported spending 21 to 40 hours per week online; 22.4% reported spending 41 to 60 hours a week online, and 19.8% reported spending over 61 hours a week on the internet. The mean number of hours per week spent online by females was 34.77 (± 26.84, range = 1–135), and, for males, this was 44.88 (± 28.46, range = 6–130). An independent group t test revealed that this difference was statistically significant, with a moderately-sized effect, *t*(503) = 4.11, *p* < 0.001, *d* = 0.366. There was a significant, but weak, positive linear relationship between age and time spent online, *F*(1,503) = 6.74, *p* < 0.05, *R* ^*2*^ = 0.013, but a stronger inverted-U quadratic relationship between these variables, *F*(1,502) = 11.10, *p* < 0.001, *R* ^*2*^ = 0.042). However, when the sample was divided into those who were currently single (N = 331), and those in some form of relationship (N = 174), there was no statistically significant difference in time spent online *t* (503) = 1.48, *p* > .10, *d* = 0.146. Similarly, there were no statically significant differences between time spent online across the different ethnic groups, *F* < 1.

The participants were also asked about the types of uses that they made of the internet, and were asked to indicate whether or not they had visited particular types of internet site during the last few months. The responses to this question are shown in Table 1, which displays the percentage of the whole sample that had visited websites of the various forms, along with percentages of male and females, and younger (less than 29 years) and older (30 years and over), participants visiting sites. In addition, Table 1 displays the Phi coefficients for these data (calculated on the actual numbers of participants, rather than the percentages displayed in Table 1). The Phi coefficients give an index of the degree of association between the variables (and are statistically significant when the corresponding chi-square statistic is significant).

**Table 1:** Percentage of sample visiting websites of various forms, along with percentage male and females, and younger and older, participants visiting sites along with Phi coefficients.

	Sample	Female	Male	Phi	<30 years	30+ years	Phi
Social Networking	95.0	97.0	92.9	.094*	96.6	91.6	.105*
Shopping/Banking	87.1	90.6	83.2	.108**	84.6	92.9	.115**
Research	82.2	83.0	81.1	.023	84.6	76.8	.094*
Gambling	70.3	70.2	70.8	.007	70.8	69.9	.012
TV and film	67.3	64.2	70.8	.071	68.0	65.8	.022
Dating & sexual	65.0	59.2	73.3	.148***	66.0	65.8	.002
News	57.4	58.5	56.2	.023	52.9	67.7	.139***
Content sharing	46.5	44.5	48.8	.042	46.0	47.7	.016
Gambling	28.3	20.0	37.5	.194***	27.4	30.3	.030
Blogging	16.8	15.5	18.3	.038	14.3	22.6	.102*
Chat rooms	9.8	1.9	16.7	.259***	6.3	14.8	.138***

*p < 0.05

**p < 0.01

***p < 0.001.

These data reveal that social networking (e.g., Facebook, Twitter) and shopping/banking websites are the most commonly used types of internet site. Gambling (including lottery sites), gaming, and sites with sexual/dating content, were used moderately often, with small numbers engaging in traditional blogging (excluding Twitter) or chat rooms. There were some gender differences in internet use, with females using social media and shopping sites more than males, and males using gaming, sexual/dating sites, and chat rooms more than females. More people under 30 years old used social networking sites, and websites for research, more often than those over 30. However, those over 30 years old used shopping/banking sites, as well as news sites, traditional blogging, and chat rooms, more often than those under 30 years old.

## Materials

### Internet Addiction Test (IAT)

The IAT [62]is a 20-item scale covering the degree to which use of internet disrupts everyday life (e.g., work, sleep, relationships, etc.). Each item is scored on a 1–4 scale, and the overall score ranges from 20 to 100. The factor structure of the IAT is currently debated [61,63], but a cut-off score of 40 or more for the total score of the IAT is taken as representing some level of problematic internet usage [7,62,64] The internal reliability of the scale has been found to be between .90 [64] and .93 [62].

### Hospital Anxiety and Depression Scale (HADS)

The HADS [65]is a widely-used measure of anxiety and depression. Originally designed for use by hospital general medical outpatients, it has been used for non-medical samples [66,67]. It contains 14 items (7 for anxiety and 7 for depression) that relate to the last week. There are 7 questions each for anxiety and depression, each question is scored from 0 to 3 depending on the severity of the symptom; the maximum score is 21 for each of the scales. Respondents can be classified into four categories: 0–7 normal; 8–10 mild; 11–14 moderate; and 15–21 severe. The test-retest reliability and validity are both strong [65], and the internal reliability is .82 for the anxiety scale, and .77 for the depression scale, for a non-clinical population [67].

### UCLA Loneliness Scale

The UCLA Loneliness Scale [68] consists of 20 statements designed to assess loneliness. Participants respond to each question using a 4-point scale (“I often feel this way”, “I sometimes feel this way”, “I rarely feel this way”, and “I never feel this way”), and each item is scored from 0 to 3, giving the total score a range from 0 to 60. A higher score indicates a higher severity of loneliness. A cut-off point for loneliness problems is usually given at one standard deviation above the mean for the sample. The scale has high reliability, with an internal consistency of .92, and a test-retest reliability of .73 [69].

### Pittsburgh Sleep Quality Index (PSQI)

**This PSQI[70]** consists of 10 main questions, some with sub-sections, where the participant is required to enter data about their sleep habits. The questionnaire gives a score of between 0 and 21, where a high score reflects worse sleep, and a score of greater than 5 reflects a poor sleeper [70]. The PSQI has been found to have a high “test-retest reliability” and a good validity when used for testing [70].

### General Health Questionnaire (GHQ-28)

The GHQ-28[71]measures a range of psychiatric and health problems, and is divided into 4 sub-scales: somatic symptoms, anxiety and insomnia, social dysfunction, and severe depression. Each sub-scale contains 7 items, all requiring a response on a 4-point Likert-type scale: *Not at all*, *No more than usual*, *Rather more than usual*, *Much more than usual*, scoring 0 to 3, respectively. The internal reliability of the scales are above .90. For the present study, only the somatic symptom scale was analysed, which asked the participants to rate the degree to which they have felt: in good general health, in need of a tonic, run down, ill, head pains, tightness or pressure in head, and hot or cold spells.

### Immune Function Questionnaire (IFQ)

The IFQconsists of 15 items that assess the frequency of various symptoms associated with poor immune function. Based on their frequency in general population, and direct relation to immune deficiencies, the following conditions were selected as a base for the items on the questionnaire: common cold [54], influenza [55], cold sores [56], pneumonia [57], sepsis [58], and skin infections [59]. Following the analysis of the major symptoms of these conditions, 19 symptom items were included on the questionnaire as signs of weakened immune system functioning: sore throat, headaches, flu, runny nose, coughing, cold sores, boils, mild fever, warts/verrucas, pneumonia, bronchitis, sinusitis, sudden high fever, ear infection, diarrhoea, meningitis, eye infection, sepsis, and long healing injuries. They were rated on a 5-point Likert-type scale (Never, Once or twice, Occasionally, Regularly, Frequently, with scores from 0 to 4 respectively). The total score ranges from 0 to 79, with high score reflecting worse immune function. The IFQ has previously been used to study the impact of stressful life events on self-reported health, such as assessing the impact of having a child with ASD. In previous work [72], the IFQ score has been found to correlate positively (*r* = .578, *p* < .001) with the number of visits to a General Medical Practitioner, there is a significant positive correlation between IFQ and a total GHQ score (*r* = .410, *p* < .01), as well as a significant correlation between IFQ and somatic symptoms sub-scale of GHQ (*r* = .493, *p* < .01).

### Procedure

All participants responded to links posted on internet sites targeted to reach a wide variety of individuals, including social network sites (e.g., Facebook, Twitter), blogging/forum pages (e.g., Mashable), gaming sites (e.g., Eurogamer.com), and internet addiction help websites. These links gave participants a brief introduction to the study, in which they were told that the research concerned the relationship between internet use and various personality and health issues. If they were interested in participating, they were instructed to follow an online link to the questionnaire. This link took the participants to a webpage containing further information about the study: again outlining that the purpose of the study was related to internet use and various personality and health issues, and which also outlined the types of questionnaires that they would be answering. The information page also gave details of their right to withdraw from the study at any time, and the steps being taken to ensure their privacy. The information was followed by a statement of consent, instructing participants only to click to begin the questionnaire if they were willing to provide consent and if they were over the age of 18. Participants were then presented with the questionnaires.

There was no time limit given for the responses to be made, and participants were given the option to save their survey and return to it at a later occasion if necessary. Once all of the questionnaires had been completed, which took participants approximately 30 min, participants were directed to a debriefing page, which thanked them for their contribution, went into further detail about the aims and purpose of the study, and provided contact details for the researcher and a counselling service, if they felt they needed any support, following issues raised within the survey. The study link remained open for three months (over the spring period), and was then closed.

### Data Analyses

Initially, the potential differences in internet addiction scores between participants with different characteristics (e.g., gender, age, etc.) were analysed using t-tests. Participants were then divided into lower- and higher-internet problem groups by using a split at the cut-off point for mild or worse internet problems based on the IAT (i.e. 40), and the association between problematic internet use scores and gender, depression, etc., was explored using chi-squared tests. The relationship between the immune function score and each of the predictor variables was explored using semi-partial correlations (to partial out the impact of the other predictors), and a stepwise regression was also employed to identify the impact of internet problem scores on immune function over and above the impact of the other predictor variables. The same analyses were also conducted for the self-report health score (GHQ). Finally, the groups were split into high and low immune function, and high and low self-reported health status (GHQ), and these groups were compared in terms of their internet addiction scores by analysis of covariance, using the other predictors as covariates. Where multiple comparisons were undertaken, more severe rejection criterion were adopted for significance testing, and effect sizes were calculated throughout.

## Results

The mean score for internet problems (IAT) for the sample was 37.25 (± 16.18, range = 0–96). The mean IAT score for females was 36.26 (± 15.36, range = 0–69), and this score for males was 38.35 (± 17.00, range = 9–96). An independent groups t-test revealed no statistically significant difference between these scores, *t* < 1, *d* = 0.006. Pearson correlations revealed a statistically significant, and moderately-sized, relationship between time spent online and the IAT score, *r*(503) = .485, *p* < .001, *R* ^*2*^ = .235, but there was no significant relationship between participants’ age and their IAT score, *r*(503) = –.025, *p* > .50, *R* ^*2*^ = .0006.

**Fig 1:**
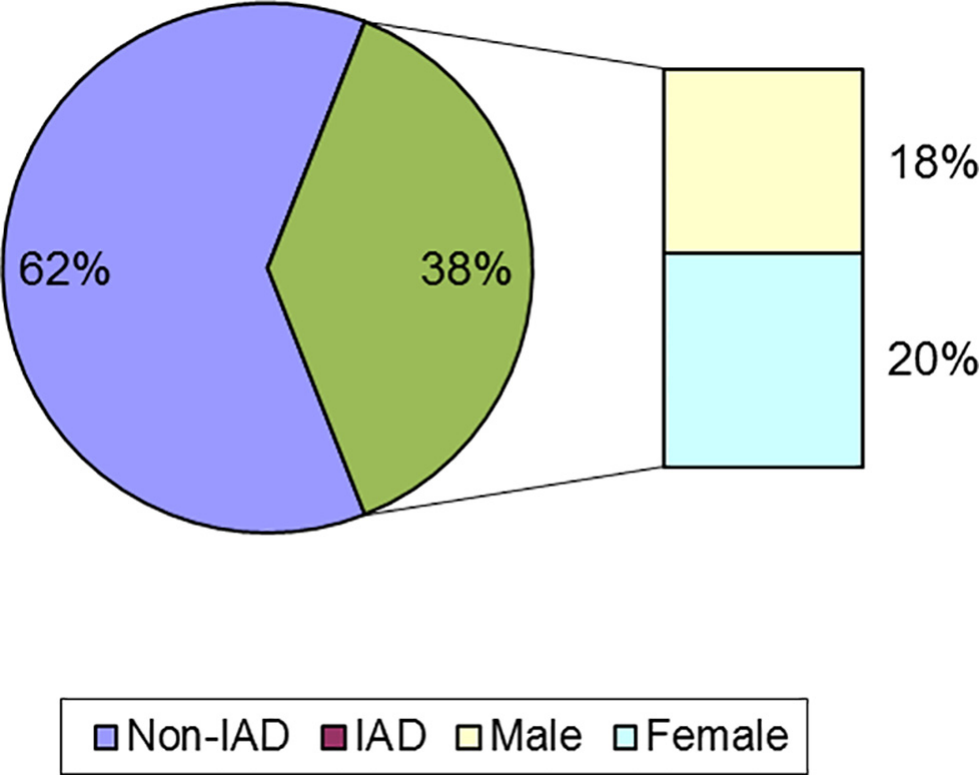
Percentage participants above and below the cut-off point for moderate or worse problematic internet usage (i.e. an IAT score of 40 or above), along with these data for females and males, separately.

The proportions of the sample falling above the cut-off point for moderate or worse problematic internet usage (i.e., an IAT score of 40 or above [62]) are shown in Fig 1 for the entire sample, along with these data for females and males, separately. Of the sample, 192 (103 female, 89 male) participants fell above the cut-off for internet problems. There was no statistically significant difference between the probability of a problematic internet use score between the genders, *chi squared* = .17, *p* > .60, *Phi* = .018. Point biserial correlations revealed no relationship between age and falling above the cut-off point, *r*_*pb*_(503) = -.002, *p* > .30, *R*_*pb*_^*2*^ = .102, although there was a statistically significant, and moderately-sized, relationship between hours spent online and falling above the cut-off point for internet addiction problems, *r*(503) = .320, *p* < .001, *R*_*pb*_^*2*^ = .102.

The top panel of Table 2 shows the sample means and standard deviations for internet problems (IAT), hours spent online, depression (HADS), anxiety (HADS), loneliness (UCLA) and sleep problems (PSQI). These means are broadly in line with those seen in previous investigations of such samples [7]. It also shows the percentage of individuals falling above the cut-off point for those scales, which, apart from sleep problems, were as expected for such a sample. Table 2 also displays the percentage of the sample with IAD falling above the cut-off for those other psychological scales. The percentages of those with an IAD also displaying a co-morbidity are higher than those for the sample as a whole. To investigate these relationships further, a series of 2x2 chi-square tests (co-morbidity present or absent versus internet problems present or absent) were conducted for each variable, and revealed that all co-morbidities were significantly associated with the presence of an internet problem: depression–*chi-square*(1) = 30.56, *p* < .001, *Phi* = .246; anxiety–c*hi-square*(1) = 38.98, *p* < .001, *Phi* = .278; loneliness–c*hi-square*(1) = 15.31, *p* < .001, *Phi* = .174; and sleep–c*hi-square*(1) = 9.38, *p* < .01, *Phi* = .136. The Pearson correlations between all of the variables, and with both somatic health problems (GHQ) and immune symptoms are also shown in Table 2, and these analyses revealed statistically significant relationships between all of the variables.

**Table 2:** Means (standard deviations) for internet problems (IAT), hours spent online, depression (HADS), anxiety (HADS), loneliness (UCLA) and sleep problems (PSQI), along with percentage of individuals falling above the cut-off point for those scales, and the percentage of people with IAD falling above the cut-off for those scales. Pearson correlations between all variables, and with somatic health problems (GHQ) and symptoms are also shown.

	Mean (SD)	% Probs	% Probs in IAD	Depression	Anxiety	Loneliness	Sleep	GHQ(S)	Symptoms
Internet problems	37.25 (16.18)			.402***	.452***	.199***	.268***	.389***	.442***
Hours online	40.64 (31.95)			.294***	.262***	.189***	.261***	.265***	.209***
Depression	4.81 (4.22)	25.0	38.5		.718***	.358***	.621***	.668***	.255***
Anxiety	4.85 (5.05)	24.4	39.6			.340***	.522***	.668***	.434***
Loneliness	20.93 (13.97)	15.5	24.0				.168***	.267***	.174***
Sleep								.562***	.387***
Stepwise regression for somatic symptoms (GHQ)					Stepwise regression for symptoms (IFQ)				
	*F*		*R* ^*2*^	SS_regression_		*F*		*R* ^*2*^	SS_regression_
Step 1^~^	(5,499) = 111.10***		.540	3442.23	Step 1^~^	(5,499) = 35.96***		.258	32944.99
Step 2^/^	(6,498) = 100.10***		.541	3422.45	Step 2^/^	(6,498) = 42.55***		.331	29628.43
Reduction	(1,498) = 2.88*		.003	19.78	Reduction	(1,498) = 55.74***		.007	3316.56

*p < 0.05

**p < 0.01

***p < 0.001

^~^Hours online, Depression, Anxiety, Sleep, Loneliness

^/^Hours online, Depression, Anxiety, Sleep, Loneliness.

The sample mean score for somatic symptoms (GHQ-S) was 7.28 (± 3.87; range = 0–19), and the mean for the immune-related symptoms questionnaire was 15.20 (± 9.43; range = 0–37). These scales had a correlation of *r* = 0.345, *p* < .001, *R* ^*2*^ = .119, with one another. The GHQ(S) score was strongly related to depression, anxiety, and sleep problems, and, to lesser extent, with the other variables. The immune-related symptoms scale was strongly related to anxiety, sleep and internet problems, and to a lesser extent with the other variables.

Given that both illness-variables (GHQ-S and IFQ) were correlated with all of the other variables, and that the IAT was related to all of the other variables, in order to explore whether internet problems (i.e. the IAT score) contributed to these illness scores, two separate stepwise multiple regressions were conducted–one for predicting the GHQ-S score, and one for predicting the IFQ score. In both cases, depression, anxiety, loneliness, sleep, and hours spent online, were entered into the regression model on the first step. All of these variables plus the internet problem (IAT) score were then entered into the model on the second step, and the degree to which the amount of variance accounted for was improved by the addition of the IAT score was calculated.

The bottom panels of Table 2 show the results for these analyses. Inspection of the data from the bottom right panel for the GHQ-S score shows that both steps of the regression were statistically significant, with the reduction in error produced by the addition of the IAT on step 2 also producing a statistically significant improvement in the prediction of the GHQ-S score. It should be noted that the improvement in prediction of the GHQ-S produced by the addition of the IAT was not very large. The same pattern of data was also found from the analysis conducted to predict the immune-related symptoms (IFQ) score. However, the addition of the IAT in step 2 produced a much larger improvement in predictive accuracy for the immune-related scores (IFQ), than it had for the somatic symptoms (GHQ-S) scores.

**Fig 2:**
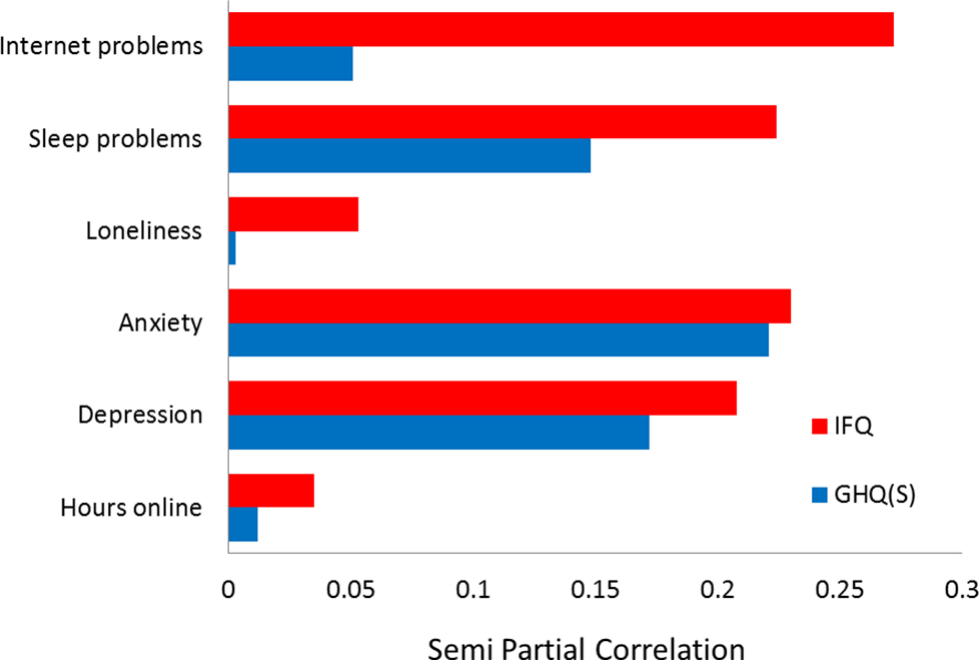
Semi-partial correlations between depression (HADS), anxiety (HADS), sleep (PSQI), loneliness (UCLA), hours online, and internet problems (IAT), and the two symptom scores (GHQ(S) and IFQ).

To further explore the nature of the relationships between the variables, the semi-partial correlations between the individual predictors (i.e. depression, anxiety, sleep, loneliness, hours online, and internet problems) and the two symptom scores (GHQ-S and IFQ) were calculated separately. The semi-partial correlations were conducted between each predictor variable and the two illness-related variables using all of the other predictor variables as co-variates. This allows the unique relationship between two variables to be observed in the absence of mediating effect of any other variables, and these values can be seen in Fig 2 for the two illness-related variables. These data show a similar pattern of relationship between the predictors and symptoms for both the GHQ-S and the IFQ; in that, depression, anxiety, and sleep problems, all had statistically significant relationships with both outcomes when the impact of the other variables were controlled. However, whereas internet problems (IAT) significantly predicted the immune-related symptoms (IFQ), this was not statistically significantly related to the GHQ(S) score.

**Fig 3:**
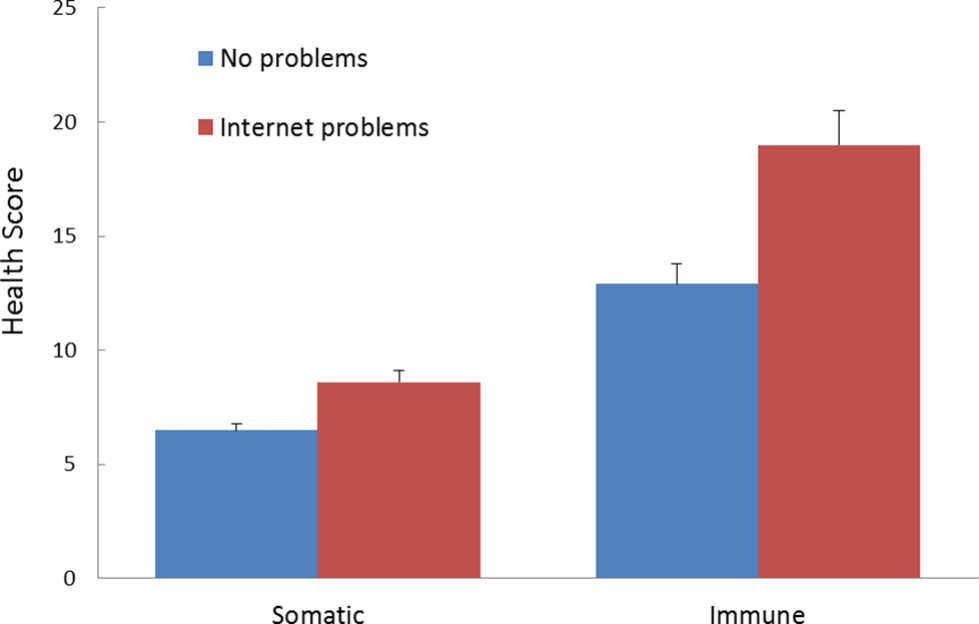
Mean general-somatic health (GHQ(S)) score (left panel), and the mean immune-related health (IFQ) score for the two IAT groups (lower and higher problems). Left panel = somatic-related scores GHQ(S); right panel = immune-related scores (IFQ).

To further explore the relationship between internet-related problems (IAT scores) and both general-somatic (GHQ-S) and immune-related (IFQ) health-problems, the sample was split into those scoring below and above the cut-off of 40 for moderate or worse internet-related problems on the IAT [62]. This created two groups: a group with no internet problems (*N* = 313; mean IAT = 26.89 + 7.89; range = 0–39), and a group with internet-problems (*N* = 313; mean IAT = 54.14 ± 11.23; range = 40–96). Fig 3 shows the mean general-somatic health (GHQ-S) score (left panel), and the mean immune-related health (IFQ) score. Inspection of the data for the GHQ-S reveals little difference between the low and high IAT groups in terms of their GHQ-S scores. These data were analysed using an analysis of covariance, with internet group as a between-subject factor, and depression, anxiety, sleep problems, loneliness, and hours online as covariates. This analysis revealed no statistically significant difference between the internet problems groups in terms of their GHQ-S scores, *F* < 1, *partial eta* ^*2*^ = .001. In contrast, the right panel of Fig 3 shows that the high internet-problems group had more immune-related health problems than the no-internet-problems group, *F*(1,498) = 27.79, *p* < .001, *partial eta* ^*2*^ = .046.

## Discussion

The current study explored the relationship between internet addiction test scores and health scores, focusing on self-assessments of immune system function as well as of general health status. This was thought an important area to investigate as there had been no previous data presented on the impact of problematic internet use on immune functioning; additionally, previous reports concerning the relationship between problematic internet usage and health-related quality of life had been discrepant with one another [9,39,40]. It was thought that the latter discrepancies might be related to the nature of the measures used to assess health status, with more psychologically-oriented health-report scales, such as the GHQ, being less related to problematic internet usage than measures more directly related to immune functioning.

Although an online recruitment strategy was adopted, the current sample had similar characteristics to many others that have previously been employed in the study of internet use. The sample was young (under 30 years old), but it did have a large age range. The average length of time spent on the internet was around 5–6 hours a day, which is in line with several current estimates [40,61]. It should be noted that this value did not differentiate between professional and personal use, and it has been suggested that this is an important in terms of internet problems [40]. However, it is unclear whether such a distinction is at all easy to make for participants. The types of activities conducted on the internet by the current participants were similar to those noted in previous studies [61]. There were gender differences in internet use. Females tended to use social media and shopping sites more than males, but males tended to use gaming, sexual/dating sites, and chat rooms, more than females. Of course, this relies on self-report data, and the differences, although statistically reliable, were small for some of these comparisons. The levels of problematic internet usage in the current sample, around 30% of the sample displayed mild or worse symptoms of internet addiction, is broadly in line with previous investigations [7].

A key finding of the current study was that self-reported problematic internet use was related to worse self-reported immune function, as indexed by numbers of immune-related symptoms. This is corroborates the results from a study which examined self-reported health-related quality of life as measured by the SF-36 and problematic internet use [40]. However, although immune function and self-reported health were related to each other, problematic internet use did not predict self-reported health symptoms, as measured by the somatic scale of the GHQ. The latter finding is in line with several previous studies that have failed to find a relationship between IAT scores and GHQ scores [9,39]. The current positive finding, in terms of the relationship between IAT scores and impaired immune function, may reflect that measuring immune-related symptoms more directly, as was done in the current study, assesses this aspect of health better than the more psychologically-oriented GHQ scale.

Notwithstanding the difficulties in the measurement of immune function that have been discussed earlier (see also below), the clinical relevance of the findings could should be placed into context given the methodological limitations of the study. The study is a correlational one, meaning that causation should not be automatically inferred from such an association. It is possible that those with greater levels of illness tend to use the internet more often than those who are fitter. However, given the ubiquity of usage of the internet, and the association between youth and internet usage, this seems unlikely, although remains a possibility that will require longitudinal research to assess. Alternatively, it could be that some third factor predicts both internet usage and poor health. However, it should also be noted that the relationship between problematic internet use and self-reported immune functioning was found to hold over and above the impact of a number of other areas of functioning (depression, anxiety, loneliness) that are associated with problematic internet use [10–12], and which are, in themselves, associated with reduced immune function [45,46,48,49]. This makes it unclear what the third mediating factor might be.

If problematic internet use did predict worse immune function, the clear question for clinicians would concern the mechanisms. One possibility is that high levels of problematic internet use have been noted to increase activation of the sympathetic nervous system [32,33]. Such an elevated sympathetic activity leads to increases in levels of nor-epinephrone and/or cortisteroids (cortisol), which, eventually, lead to decreased immune function [52]. Thus, this route may well underlay the relationship between problematic internet use and reduced immune function, but will require further investigation. The latter suggestion has some relevance for the future conceptualisation and exploration of the clinical features of problematic internet usage.

The relationship between IAT scores and immune function reflects the fact that the overall usage of the internet for some people is regarded, by themselves, as a problem–however, what they are using the internet for will differ between these individuals. For example, the current study found gender differences in the uses that people had for the internet, and it may be that particular usages are related to reduction in immune function differentially between the genders. Further detailed work regarding the type of internet usage, such as the exact nature of the use, and the time spent online for professional and personal use, may shed further light on the relationship between internet use and reductions in immune function.

As always, there are some limitations to the current study that need to be noted. The current sample was recruited online, and this may have biased the type of individual who participated in the study. However, it should be mentioned that the range of individuals in the sample was quite wide in terms of their ages, and their other characteristics, and the sample seemed to be in line with those used in previous studies. It should be noted that the current study did not distinguish between professional and personal use of the internet, which may be important to examine. For example, the level of compulsion and urgency to use the internet may impact on stress levels to a greater extent than hours that have to be spent on the internet for work. That is, a distinction might be made between those who work hard and are stressed for that reason, and people who have an internet problem and are stressed and unwell because of this problem.

In terms of the potential alternative predictors of the reduced immune function seen in high problem users, future work might consider the role of multiple addictions that could have affected the group of problem internet users. Information concerning pharmacological and non-pharmacological addiction was not collected in the current report, and this might covary with internet problems, and affecting the immune function. Similarly, recent stressful life events might have impacted addictive behaviour and immune system function, as could the social conditions of the participants. Both of these aspects could be examined by further research.

The reliance on self-report for immune function may be subsequently be bolstered by the use of analysis of blood cells, which would add support to the current conclusions. However, as noted above, there is not a perfect relationship between the physiology of immune function and the experience of symptoms [54], and self-report of colds and flus is taken as valid measure of immune function in this regard [31,44]. Certainly, it has been found that self-reports of illness symptoms–especially regarding upper respiratory infections (e.g., colds and flu), as used in the current study, correlates well with objective immunoglobin readings [73].

Finally, it should be acknowledged that although the current study did show relationships between problematic internet use and immune-related symptoms, there are two caveats to drawing causal conclusions from this association that should be mentioned. Firstly, as the study was not longitudinal in nature, then causal inference should not be taken to be proven. Secondly, as many of the predictor variables were correlated with one another, then this might have produced a degree of co-linearity in the regression analyses making interpretation difficult. Although it should be noted that the use of semi-partial correlations does, to some extent, ameliorate this difficulty.

In summary, the current report established a link between problematic internet use and reporting of greater numbers of symptoms associated with a reduced immune-system function. This relationship was independent of the numbers of hours spent online, and also of the impact of any co-morbid symptoms of problematic internet use, such as depression, isolation, and anxiety. It was suggested that the negative impact of immune function may be mediated by heightened stress, and also by the increased sympathetic nervous activity that is sometimes displayed by internet addicts.
