# Correction: Problematic Internet Usage and Immune Function

**DOI:** 10.1371/journal.pone.0140692

**Published:** 2015-10-09

**Authors:** Phil Reed, Rebecca Vile, Lisa A. Osborne, Michela Romano, Roberto Truzoli

There are errors in the author affiliations. The affiliations should appear as shown here:

Phil Reed^1^, Rebecca Vile^1^, Lisa A. Osborne^2^, Michela Romano^3^, Roberto Truzoli^3^

**1** Swansea University, Swansea, United Kingdom, **2** Abertawe Bro Morgannwg University Health Board, Swansea, United Kingdom, **3** Università degli Studi di Milano, Milan, Italy.

There is an error in Table 1. The second occurrence of the word “Gambling” in the first column should read “Gaming.” Please see the corrected Table 1 here.

**Table 1:** Percentage of sample visiting websites of various forms, along with percentage male and females, and younger and older, participants visiting sites along with Phi coefficients.

	Sample	Female	Male	Phi	<30 years	30+ years	Phi
Social Networking	95.0	97.0	92.9	.094*	96.6	91.6	.105*
Shopping/Banking	87.1	90.6	83.2	.108**	84.6	92.9	.115**
Research	82.2	83.0	81.1	.023	84.6	76.8	.094*
Gambling	70.3	70.2	70.8	.007	70.8	69.9	.012
TV and film	67.3	64.2	70.8	.071	68.0	65.8	.022
Dating & sexual	65.0	59.2	73.3	.148***	66.0	65.8	.002
News	57.4	58.5	56.2	.023	52.9	67.7	.139***
Content sharing	46.5	44.5	48.8	.042	46.0	47.7	.016
Gaming	28.3	20.0	37.5	.194***	27.4	30.3	.030
Blogging	16.8	15.5	18.3	.038	14.3	22.6	.102*
Chat rooms	9.8	1.9	16.7	.259***	6.3	14.8	.138***

*p < 0.05

**p < 0.01

***p < 0.001.
